# Role of androgens in dhea-induced rack1 expression and cytokine modulation in monocytes

**DOI:** 10.1186/s12979-016-0075-y

**Published:** 2016-05-29

**Authors:** Emanuela Corsini, Valentina Galbiati, Angela Papale, Elena Kummer, Antonella Pinto, Melania M. Serafini, Antonio Guaita, Roberto Spezzano, Donatella Caruso, Marina Marinovich, Marco Racchi

**Affiliations:** Laboratory of Toxicology, Department of Pharmacological and Biomolecular Sciences (DiSFeB), Università degli Studi di Milano, Milan, Italy; Department of Drug Sciences - Pharmacology Unit, University of Pavia, Viale Taramelli 14, Pavia, 27100 Italy; “Golgi Cenci” Foundation, Abbiategrasso, Italy; Mass Spectrometry Laboratory “Giovanni Galli”, DiSFeB, Università degli Studi di Milano, Milan, Italy

**Keywords:** Immunosenescence, PKC, Cytokines, Androgens, Signal transduction, Monocytes

## Abstract

**Background:**

Over the past fifteen years, we have demonstrated that cortisol and dehydroepiandrosterone (DHEA) have opposite effects on the regulation of protein kinase C (PKC) activity in the context of the immune system. The anti-glucocorticoid effect of DHEA is also related to the regulation of splicing of the glucocorticoid receptor (GR), promoting the expression of GRβ isoform, which acts as a negative dominant form on GRα activity. Moreover, it is very well known that DHEA can be metabolized to androgens like testosterone, dihydrotestosterone (DHT), and its metabolites 3α-diol and 3β-diol, which exert their function through the binding of the androgen receptor (AR). Based on this knowledge, and on early observation that castrated animals show results similar to those observed in old animals, the purpose of this study is to investigate the role of androgens and the androgen receptor (AR) in DHEA-induced expression of the PKC signaling molecule RACK1 (Receptor for Activated C Kinase 1) and cytokine production in monocytes.

**Results:**

Here we demonstrated the ability of the anti-androgen molecule, flutamide, to counteract the stimulatory effects of DHEA on RACK1 and GRβ expression, and cytokine production. In both THP-1 cells and human peripheral blood mononuclear cells (PBMC), flutamide blocked the effects of DHEA, suggesting a role of the AR in these effects. As DHEA is not considered a direct AR agonist, we investigated the metabolism of DHEA in THP-1 cells. We evaluated the ability of testosterone, DHT, and androstenedione to induce RACK1 expression and cytokine production. In analogy to DHEA, an increase in RACK1 expression and in LPS-induced IL–8 and TNF–α production was observed after treatment with these selected androgens. Finally, the silencing of AR with siRNA completely prevented DHEA-induced RACK1 mRNA expression, supporting the idea that AR is involved in DHEA effects.

**Conclusions:**

We demonstrated that the conversion of DHEA to active androgens, which act via AR, is a key mechanism in the effect of DHEA on RACK1 expression and monocyte activation. This data supports the existence of a complex hormonal balance in the control of immune modulation, which can be further studied in the context of immunosenescence and endocrinosenescence.

## Background

In early studies aimed to characterize the molecular mechanisms underlying immunosenescence, we identified a defective protein kinase C (PKC) activation central to the function of immune cells. In particular, we demonstrated that the failing element in PKC activation was the reduced expression of its scaffold protein Receptor for Activated C Kinase 1 (RACK1), which underlies the functional impairment of immune system associated with aging [1–5]. RACK1 is a 36–kDa protein that contains seven WD-domain motifs and is related to G protein β subunits [6, 7]. RACK1 is a highly conserved intracellular adaptor protein, which was originally identified as the anchoring protein for activated PKC [8, 9]. In the past 20 years, the number of binding partners and validated cellular functions for RACK1 has increased. It is believed to act as signal integrator, which interconnects distinct signaling pathways to control many essential cellular processes, including protein translation, developmental processes, multiple hormonal responses, pathogen infection resistance, environmental stress responses, and miRNA production. These multiple functional roles are fitting, considering the scaffolding nature of RACK1 protein [10–14].

Relative to PKC, RACK1 is able to interact preferentially with PKCβII [8] and PKCε [9]. RACK1 stabilizes their active conformation and promotes their translocation to specific substrates in order to activate defined pathways [6, 7]. Using antisense oligonucleotides to decrease RACK1 expression, or using a PKCβ pseudosubstrate to selectively inhibit PKCβ activation, a significant reduction in LPS-induced monocytes/macrophages activation was observed, indicating the involvement of RACK1 and PKCβ in the signal transduction pathways triggered by LPS [1, 15].

The role of hormones in the control of RACK1 expression was suggested by a series of experimental evidence. While in old rats there is evidence that RACK1 expression and LPS-stimulated production of tumor necrosis factor-α (TNF α) is reduced in alveolar macrophages, we demonstrated that castration of young male rats produced effects on alveolar macrophages similar to those of aging. Conversely, the supplementation of dehydroepiandrosterone (DHEA) to old rats restored the age-decreased level of RACK1, LPS-stimulated production of TNF α in alveolar macrophages, and mitogen-induced splenocyte proliferation, suggesting that immunosenescence is partially under hormonal control and can be restored by appropriate replacement therapy [2].

Similarly, we also observed a direct correlation between circulating DHEA and RACK1 expression in human peripheral blood leukocytes. In vitro treatment with physiological concentrations of DHEA resulted in increased RACK1 expression in leukocytes and lymphocyte proliferation, confirming the role of this hormone in the modulation of RACK1 expression and immune functions [3].

DHEA is the most abundantly secreted adrenal steroid in humans. It is known to increase throughout childhood and puberty, and then to decrease with old age. The average serum DHEA concentration in men aged 25–34 is 15.9 + 6.1 nM but falls to 5.4 + 1.7 nM in those aged 75–85 [16]. Reduced secretion of DHEA during aging has been related to a series of age-associated conditions, including atherosclerosis and cardiovascular disease, breast cancer, obesity, loss of muscle mass, and diabetes; there is a view that supplementation with DHEA may have a number of significant clinical uses [17].

It is well documented that DHEA affects multiple cellular functions of the endocrine, immune, and nervous systems. However, despite intense effort by scientists to elucidate the multifunction of DHEA, its mechanism of action is still elusive. Most of its physiological actions have been attributed to its conversion to either androgens or estrogens. Depending on the tissue, DHEA can be indeed metabolized to 4-androstenedione, 5α-androstenedione, testosterone, estrogen, and other biologically active steroids [18, 19].

More recently, we demonstrated in THP-1 cells that DHEA can antagonize physiological functions of endogenous glucocorticoid [20] inducing a dose-related up-regulation of GRβ, through a modulation in the expression of the splicing-related protein SRp30c. Moreover, GRβ knockdown prevented DHEA-induced RACK1 expression and modulation of cytokine release [21]. This data indicated the ability of GRβ to act as a dominant-negative inhibitor of GRα function, which could underlie the anti-glucocorticoid effect of DHEA on RACK1 protein expression.

Glucocorticoid, progestin, and androgen hormones function via their specific receptors, which recognize a common DNA binding site: the glucocorticoid response element (GRE) or hormone response element (HRE). HRE can function as a response element for all steroid classes except for estrogens, allowing extensive crosstalk between receptors and shared target genes [22, 23]. To shed light on the mechanisms underlying DHEA-induced RACK1 expression and cytokine production in monocytes, we investigated the role of androgens and androgen receptor on DHEA effects. Collectively, our findings demonstrate that, similarly to DHEA, other androgens exerted stimulatory effects on immune cells, and AR silencing completely prevented DHEA-induced RACK1, supporting the notion that DHEA conversion to androgens and AR are central to the biological effects of DHEA in monocytes. This data further contributes to our understanding of the mechanism of action of DHEA.

## Methods

### Chemicals

Four-Androstene-3, 17-dione (androstenedione), DHEA, DHT, 3α-DIOL, 3β-DIOL, flutamide (Flut), testosterone were obtained from Sigma Aldrich (St Louis, MO, USA). Steroids were dissolved in dimethyl sulfoxide (DMSO) at 50 mM and further diluted (the final concentration of DMSO in culture medium was 0.2 %). Lipopolysaccharide from *Escherichia coli* serotype 0127:B8 (LPS) was from Sigma Aldrich. All cell culture reagents and supplements were from Sigma. Antibodies against RACK1 and AR were from BD Biosciences (Franklin Lakes, NJ, USA), GRβ from Abcam (Cambridge, UK), and anti-human β-actin from Sigma Aldrich. AR siRNA and scrambled controls were from Cell Signaling Technology (Danvers, MA, USA). Electrophoresis reagents were from Bio-Rad (Hercules, CA, USA). All reagents were purchased at the highest purity available.

### Cells

THP-1 cells, obtained from Istituto Zooprofilattico di Brescia (Italy), were diluted to 10^6^ cells/mL in RPMI 1640 without phenol red containing 2 mM L-glutamine, 0.1 mg/mL streptomycin, 100 IU/mL penicillin, gentamycin 10 μg/ml, 50 μM 2–mercaptoethanol, supplemented with 5 % heated-inactivated dialyzed fetal calf serum (culture media) and cultured in suspension at 37 °C in 5 % CO_2_ incubator. Cells were treated as reported in the legend to figures.

### Human volunteers and ethical statement

Peripheral blood mononuclear cells (PBMCs) were isolated by Ficoll gradient centrifugation from fresh blood of human volunteers. Five healthy females (23–52 year-old) were selected according to the guidelines of the Italian Health authorities, and to the Declaration of Helsinki principles, within a protocol approved by the Ethics Committee of the University of Pavia (NCT01345110), and signed an informed consent. Following centrifugation at 900 g at 25 °C for 30 min with no brake, the PBMCs layer was removed and washed twice in Hanks’ balanced salt solution (HBSS). PBMCs were resuspended at 10^6^/ml in culture media and treated as reported in the legend to figures.

### Cell viability

Cytotoxicity was assessed by leakage of lactate dehydrogenase (LDH). LDH activity was determined in cell-free supernatants using a commercially available kit (Takara Bio Inc., Japan). Results are expressed as optical density (OD).

### Real time RT-PCR

Total RNA was isolated at different times of treatment using a commercially available kit (TriReagent from Sigma) following the supplier’s instructions. For the synthesis of cDNA, 2.0 μg of total RNA was retro-transcribed using the High-Capacity cDNA Archive Kit from Applied Biosystems (Foster City, CA, USA) following the supplier’s instructions. RACK1 gene expression was evaluated by real-time reverse transcription-polymerase chain reaction (Real-time PCR). For PCR-analysis, Taq-ManTM-PCR technology was used. PCRs were performed in duplicate and according to the standard protocol suggested by the manufacturer. For each PCR reaction, 10 ng of total RNA were used. The 18S ribosomal RNA transcription was used as endogenous reference, and the quantification of the transcripts was performed by the 2^-ΔΔCT^ method [24].

### Western blot analysis

The presence of RACK1 and AR in cell homogenates was assessed by Western blot analysis. Briefly, cells (3x10^6^) were treated as described in the legends. At the end of treatments, cells were collected, washed once with PBS, centrifuged and lysed in 100 μl of homogenization buffer (50 mM TRIS, 150 mM NaCl, 5 mM EDTA pH 7.5, 0.5 % Triton X-100, 50 μM PMSF, 2 μg/mL aprotinin, 1 μg/mL pepstatin and 1 μg/mL leupeptin) and denatured for 10 min at 100 °C. The protein content of the cell lysate was measured using a commercial kit (Bio-Rad). 5 μg (for RACK1) or 10 μg (for GRβ and AR) of extracted proteins were electrophoresed in a 12 % SDS-polyacrylamide gel under reducing conditions. The proteins were then transferred to PVDF membrane (Amersham, Little Chalfont, UK). The proteins were visualized using primary antibodies for RACK1 (1:1000), GRβ (1:1000), AR (1:1000) and β–actin (1:5000) and developed using enhanced chemiluminescence (Pierce, Thermo Scientific, Rockford, IL, USA). The image of the blot was acquired and analyzed with the Molecular Imager Gel Doc XR (BioRad).

### Cytokine production

Cytokine production was assessed in cell free supernatants by specific sandwich ELISAs, commercially available (R&D System, Minneapolis, MN, USA). Cell-free supernatants obtained by centrifugation at 1200 rpm for 5 min were stored at −20 °C until measurement. Limits of detection were 15 pg/ml. Results are expressed in pg/ml.

### Quantitative analysis of steroids by LC-MS/MS

Intracellular testosterone and its metabolites DHT, 3α–DIOL and 3β–DIOL were assessed by LC-MS/MS as previously described [25]. Cells (1 × 10^7^) were treated with DHEA 10 nM or DMSO as vehicle control for 1 h, washed twice with PBS and lysed. Samples, added with 13C internal standards, were extracted and purified with 1 ml of MeOH/acetic acid (99:1, v/v). The organic phases were passed through an SPE cartridge, steroids fraction concentrated and transferred into auto-sampler vials before LC–MS/MS analysis. Quantitative analysis was performed on the basis of calibration curves prepared and analyzed as described above for samples. Positive atmospheric pressure chemical ionization (APCI) experiments were performed using a linear ion trap-mass spectrometer (LTQ, ThermoElectron Co., San Jose, CA, USA) and the LC mobile phases were (A) H2O/0.1 % formic acid and (B) methanol (MeOH)/0.1 % formic acid. The gradient (flow rate 0.5 ml/min) was described previously [25].

### Small interference RNA (siRNA) for AR

To evaluate the role of AR in DHEA-induced RACK1 expression, silencing experiments were conducted. The effect of inducing RNA interference on AR was assessed using commercially available reagents (SignalSilence™ siRNA from Cell Signaling Technology) following the manufacturer’s instructions. As control, siRNA (scr), an siRNA sequence that will not cause the specific degradation of any cellular messages was used. Forty-eight hours after siRNA transfection, Western blot analysis using AR antibody was performed to confirm silencing in whole cell lysates. Cells were then adjusted to 10^6^/ml and treated with DHEA (10 nM) or DMSO as vehicle control for 16 h to assess RACK1 mRNA expression and 24 h to assess RACK1 protein expression.

### Statistical analysis

All experiments were performed at least three times, with representative results shown. Data are expressed as mean ± standard deviation (SD). Statistical analysis was performed using GraphPad InStat version 3.0a for Macintosh (GraphPad Software, San Diego, CA, USA). Differences were considered significant at *p* ≤ 0.05.

## Results

### The anti-androgen flutamide prevents the stimulatory effects of DHEA on RACK1 expression and cytokine production

**Fig. 1:**
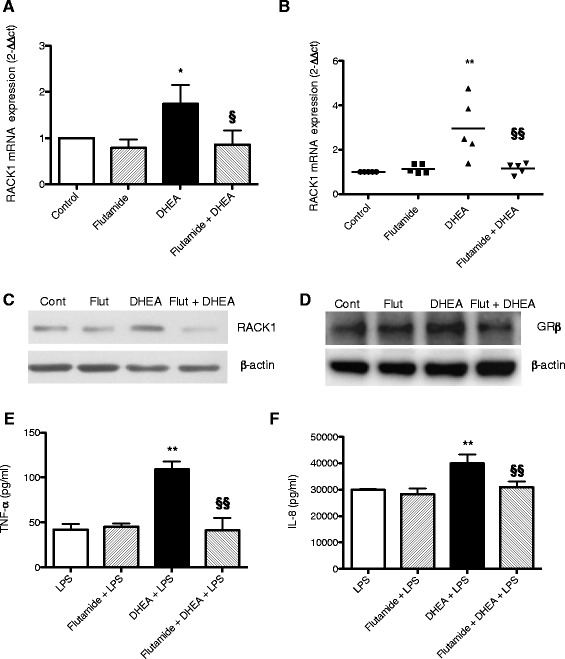
The anti-androgen flutamide prevents DHEA-induced effects. **a**, **b** RACK1 mRNA expression. THP-1 cells (**a**) or PBMC (**b**) were treated with flutamide (50 μM) or DMSO for 1 h, followed by DHEA (10 nM) or DMSO for 16 h. DMSO (0.1 % final concentration) was used as vehicle control in all experiments. RACK1 mRNA expression was assessed by Real Time-PCR. Results are expressed as mean ± SD of three independent experiments (**a**) or as dots of individual donors responses (**b**). *p < 0.05 vs Control cells and §*p* < 0.05 vs DHEA alone. **c**, **d** Representative Western blot analyses of RACK1 (**c**) or GRβ (**d**) immunoreactivity in THP-1 cells. β-Actin immunoreactivity was used as protein loading control. Representative Western blots are reported. Cells were treated with flutamide (Flut, 50 μM) or DMSO (Cont) for 1 h and then DHEA (10 nM) or DMSO was added for 24 h. **e**, **f** Effect on cytokine production. THP–1 cells were treated with flutamide (50 μM) for DMSO or 1 h, and then DHEA (10 nM) or DMSO was added. After 24 h, LPS (10 ng/ml) was added for 3 h to assess TNF-α release (**e**) or 24 h to assess IL–8 release (**f**). Results are expressed as mean ± SD, *n* = 3 replicates. Data is representative of three independent experiments. Statistical analysis was performed with Tukey’s multiple comparison test with **p < 0.01 vs LPS treated cells and §§, *p* < 0.01 vs DHEA + LPS treated cells

The aim of this study was to investigate the role of androgens and AR in the immunostimulatory effects of DHEA. To address this question, we first investigated the ability of the anti-androgen flutamide to modulate the stimulatory effects of DHEA in both the human promyelocytic THP-1 cell line, and in human peripheral blood mononuclear cells (PBMCs). THP-1 is a human leukemia monocytic cell line derived from the peripheral blood of a 1-year-old human male with acute monocytic leukemia. This cell line has been extensively used to study monocyte/macrophage functions, mechanisms, and signaling pathways [26]. PBMCs were obtained from five adult healthy females (28-year-old) and were used to support the relevance of data obtained in THP-1 cells and to exclude possible gender effects. THP-1 cells and PBMCs were treated for 1 h with flutamide (50 μM), then a physiologically relevant concentration of DHEA (10 nM) or DMSO as vehicle control was added. After 16 h of treatment, Real Time-PCR was used to assess RACK1 mRNA expression. In both experimental models, flutamide completely prevented DHEA-induced RACK1 mRNA expression (Fig. 1a and b), suggesting the role of AR in the effect of DHEA on RACK1 expression.

The rest of the data presented was conducted using THP-1 cells. Next, the effect of flutamide on DHEA-induced RACK1 protein expression and cytokine production was investigated. Cells were treated with DHEA for 24 h as optimal time for RACK1 protein expression [27]. After DHEA treatment, LPS (10 ng/ml) was added for 3 and 24 h to assess TNF-α and IL-8 release, respectively. Flutamide not only prevented DHEA-induced RACK1 mRNA expression, but also RACK1 protein expression as assessed by Western blot analysis (Fig. 1c), and cytokine production as assessed by LPS (10 ng/ml)-induced TNF-α and IL-8 release (Fig. 1e and f). Similar inhibitory effects on LPS-induced cytokine production were observed in human PBMC treated with DHEA in the presence of flutamide (data not shown). The release of TNF-α and IL-8 in DMSO treated cells was below the detection limit of the ELISA (data not shown). The inhibitory effect of flutamide was not due to cytotoxicity, as assessed by identical LDH leakage in control and treated cells: in control cells the optical density (OD) value was 0.582 ± 0.023 vs 0.597 ± 0.017 in flutamide treated cells.

Furthermore, considering the anti-glucocorticoid effect of DHEA, mediated by the induction of GRβ [21], we demonstrate here that flutamide also prevents DHEA-induced GRβ protein expression (Fig. 1d). This data clearly indicates that AR is central to the action of DHEA, and confirms early data obtained in experimental animals on the role of androgens in the maintenance of RACK1 expression and immune function [2].

### DHEA is metabolized to androgens in THP-1 cells, and other androgens have a similar effect to that of DHEA

**Fig. 2:**
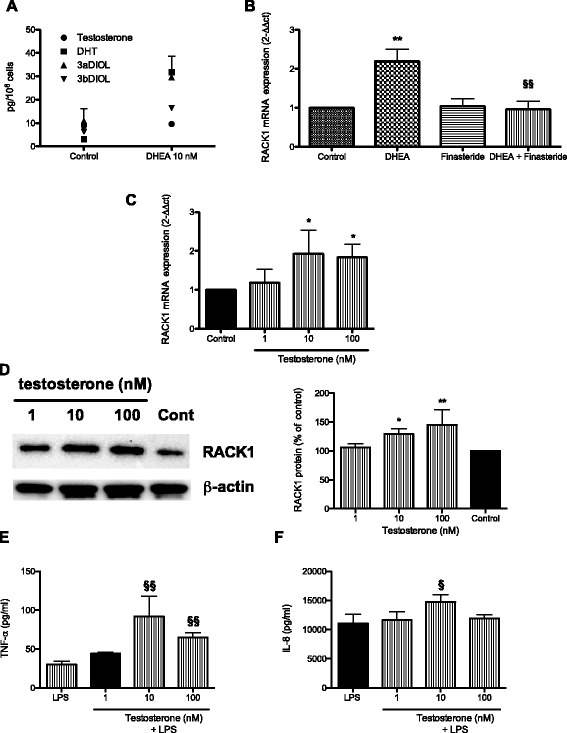
DHEA is metabolized to DHT in THP–1 cells; and testosterone (similarly to DHEA) affects RACK1 expression. **a** DHEA metabolism. THP–1 cells (10^7^) were treated with (10 nM) or DMSO as vehicle control for 1 h. Testosterone, DHT, and its metabolites 3α-diol and 3β-diol were assessed by LC-MS/MS in cell lysates. Each dot represents the mean ± SD of 4 samples obtained in two independent experiments. **b** The 5α-reductase inhibitor finasteride blocks DHEA-induced RACK1 expression. THP-1 cells were treated with finasteride (0.1 μM) for 1 h and then DHEA (10 nM) or DMSO as vehicle control was added for 16 h. RACK1 mRNA expression was assessed by Real Time-PCR. Results are expressed as mean ± SD of three independent experiments, with §*p* < 0.05 vs DHEA alone. **c**, **d** Testosterone induces a dose-related increase in RACK1 expression. THP-1 cells were treated with increasing concentrations of testosterone (1–100 nM) or DMSO as vehicle control for 16 h for mRNA expression (**c**) or 24 h for protein expression (**d**). RACK1 mRNA expression was assessed by Real Time-PCR. Results are expressed as mean ± SD of four independent experiments, with *p < 0.05 vs Control cells. RACK1 immunoreactivity was assessed by Western blot analysis of cell lysates. β-Actin immunoreactivity was used as protein loading control and to normalize RACK1 expression. A representative Western blot is reported together with a densitometric analysis of data obtained from three independent experiments. Results are expressed as mean ± SD, with *p < 0.05 vs Control cells. **e**, **f** Effect of testosterone on LPS-induced cytokine production. THP-1 cells were treated with increasing concentrations of testosterone (1–100 nM) or DMSO as vehicle control. After 24 h, LPS (10 ng/ml) was added for 3 h to assess TNF-α release (**e**) or 24 h to assess IL–8 release (**f**). Results are expressed as mean ± SD, *n* = 3 replicates. Data is representative of three independent experiments. Statistical analysis was performed with Tukey’s multiple comparison test with §*p* < 0.05 and §§*p* < 0.01 vs LPS treated cells

**Fig. 3:**
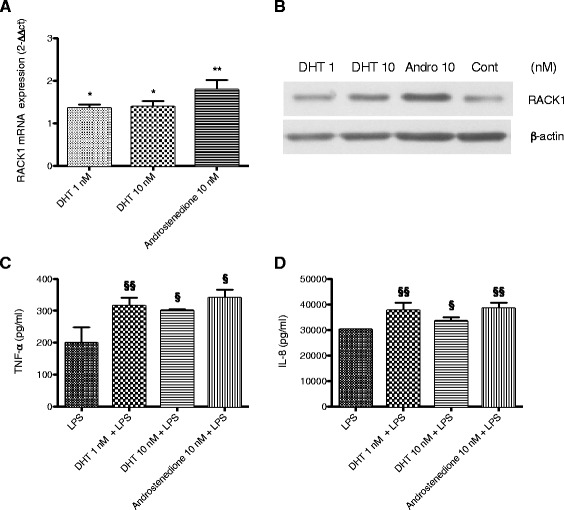
DHT and androstenedione, similarly to DHEA, affect RACK1 expression. **a**, **b** Increased RACK1 expression following treatment with DHT and androstenedione. THP-1 cells were treated with increasing concentrations of DHT (1–10 nM), androstenedione (10 nM) or DMSO as vehicle control for 16 h for mRNA expression (**a**) or 24 h for protein expression (**b**). RACK1 mRNA expression was assessed by Real Time-PCR. Results are expressed as mean ± SD of three independent experiments, with *p < 0.05 and **p < 0.01 vs Control cells. RACK1 immunoreactivity was assessed by Western blot analysis of cell lysates (**b**). A representative Western blot of RACK1 immunoreactivity in cell lysate is shown. **c**, **d** Effect of DHT and androstenedione on LPS-induced cytokine production. THP–1 cells were treated with increasing concentrations of DHT (1–10 nM), androstenedione (10 nM) or DMSO as vehicle control. After 24 h, LPS (10 ng/ml) was added for 3 h to assess TNF-α release (**c**) or 24 h to assess IL–8 release (**d**). Results are expressed as mean ± SD, *n* = 3 replicates. Data is representative of three independent experiments. Statistical analysis was performed with Tukey’s multiple comparison test with §*p* < 0.05 and §§*p* < 0.01 vs LPS treated cells

Most of DHEA physiological actions have been attributed to its conversion to either androgens or estrogens, therefore, we investigated the ability of THP-1 cells to metabolize DHEA. THP-1 cells were treated for 1 h with DHEA (10 nM) or DMSO as vehicle control. Testosterone, DHT, and its metabolites 3α-diol and 3β-diol were evaluated by LC-MS/MS in cell lysates (Fig. 2a). Data indicates the ability of THP-1 cells to rapidly convert DHEA to androgens. This finding prompted us to investigate the ability of the androgens testosterone, DHT, and 4-androstenedione, the first intermediate of DHEA in the synthesis of testosterone, to induce RACK1 expression and cytokine production. In analogy to DHEA, an increase in RACK1 expression (both mRNA and protein) and in LPS-induced IL-8 and TNF-α production was observed after treatment with these androgens (Figs. 2 and 3). The role of DHT in DHEA-induced RACK1 expression was also corroborated by the ability of finasteride, a 5α-reductase inhibitor, to completely block the effect of DHEA on RACK1 mRNA expression (Fig. 2b).

In details, THP-1 cells were treated with increasing concentrations of testosterone (1–100 nM). These concentrations are physiologically relevant, as the ranges of plasma levels of testosterone in female are 0.7-3 nM, and 10–50 nM in male. Testosterone was able to induce a dose-related increase of RACK1 mRNA expression (Fig. 2c), and protein (Fig. 2d), which was associated with a statistically significant increase in the response to LPS (10 ng/ml) as assessed by TNF-α and IL-8 release (Fig. 2e). 10 nM testosterone was the most effective concentration.

As shown in Fig. 3, THP-1 cells were treated with DHT (1 and 10 nM) and 4-androstenedione (10 nM) for 16 h to assess the effect on mRNA expression (Fig. 3a), or 24 h for protein expression (Fig. 3b). Following 24 h of treatment with DHT or 4-androstenedione, LPS (10 ng/ml) was added for 3 and 24 h to assess TNF-α and IL-8 release, respectively (Fig. 3c and d). Both DHT and 4-androstenedione were able to significantly increase RACK1 mRNA and protein expression, as well as the response to LPS. Compared to testosterone, DHT appears to be more potent: at 1 nM the increase in RACK1 expression and in LPS-induced cytokine production was already statistically significant, whereas testosterone 1 nM was ineffective (Fig. 2). This is consistent with the notion that DHT is more potent than testosterone.

**Fig. 4:**
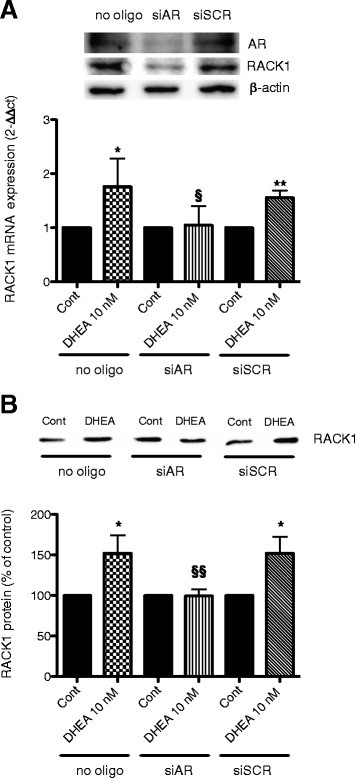
AR silencing prevents DHEA-induced RACK1 expression. THP-1 cells were transfected with transfection reagent alone (no oligo) or with siRNA (60 nmol) targeted to AR (siAR) or with control siRNA (scr). After 48 h, extracts were prepared for Western blot analysis of AR, RACK1 and β-actin (loading control) immunoreactivity to estimate AR knockdown efficiency (inset). After silencing, cells were incubated with DHEA (10 nM) or DMSO as vehicle control for 16 h to assess mRNA expression (**a**) and 24 h to assess protein expression (**b**). Results are expressed as mean ± SD of three independent experiments, with *p < 0.05 and **p < 0.01 vs control cells, and §*p* < 0.05 vs no oligo or siSCR treated cells

Finally, to confirm that AR is necessary to mediate DHEA-induced RACK1 expression, silencing experiments were conducted. As shown in Fig. 4, AR silencing completely prevented DHEA-induced RACK1 mRNA and protein expression, supporting the notion that AR is indeed involved in DHEA effects. Additional confirmation of the link between AR and the expression of RACK1 came from the observation that the silencing of AR is associated with a reduction in RACK1 immunoreactivity, as assessed by Western blot analysis of cell lysates following 48 h of silencing (Fig. 4).

## Discussion

DHEA has been reported to have several beneficial effects in aging humans [17, 19, 28], and it is therefore of interest to determine the mechanisms behind these diverse effects, which are still incompletely understood. In addition, with the current utilization of DHEA as a dietary supplement, the mechanism of action of this sterol and its metabolites is important to study. The main purpose of this study was to shed light on the mechanism underlying the effect of DHEA on RACK1 expression and on the activation of innate immune cell, as well as to reconcile data we have accumulated over the last fifteen years [2, 21]. In the present manuscript, our results indicate that the activation of AR is central to the action of DHEA on RACK1 expression and cytokine release. In THP-1 cells, we found that DHEA is rapidly converted to DHT and downstream metabolites. We demonstrated that the effect of DHEA and its metabolites on RACK1 expression could be completely prevented by using flutamide as an AR antagonist, blocking the expression of the receptor by siRNA, or using finasteride to block DHT synthesis, strongly suggesting a pivotal role of androgens and AR in RACK1 modulation. Overall, these findings contribute to our understanding of the physiological role of hormones in monocyte functions, and support the use of DHEA to manipulate monocyte activation and to counteract immunosenescence. However, the need for large, long-term placebo controlled clinical trials to demonstrate safety and efficacy of DHEA is still an open question [17].

We have previously demonstrated that cortisol and DHEA have opposite effects on the regulation of PKC activity involved in immune processes such as cytokine release and lymphocyte proliferation [2, 27]. Physiological concentrations of cortisol exert an inhibitory effect on RACK1 expression through the presence of a glucocorticoid receptor (GR)-responsive sequence on the promoter region of the human guanine nucleotide-binding protein β-2-like 1 (GNB2L1) gene, which codes for RACK1 protein [29]. Conversely, treatment with DHEA could restore the levels of RACK1 protein both in vivo and in vitro [2]. In the context of the immune system, we demonstrated that the anti-glucocorticoid effect of DHEA on RACK1 expression, and the related PKC signaling, may be due to the influence of DHEA on alternative splicing of the mRNA coding for the human GR in favor of the expression of the β isoform [21]. In the current manuscript, we show that by blocking AR using flutamide, DHEA-induced GRβ protein expression could be completely prevented, positioning AR at the center of the action of DHEA. In both human PBMC and THP-1 cells, flutamide prevented DHEA-induced RACK1 mRNA expression and LPS-induced cytokine production. Similarly, blocking the expression of the receptor by the use of siRNA, the actions of DHEA on the same mechanisms was impaired.

DHEA mediates its action via several signaling pathways involving specific membrane receptors. These pathways and receptors include the G-coupled protein receptors demonstrated in bovine aortic endothelial cells and human umbilical vein endothelial cells, the σ1-receptor in neurons, and via transformation into androgen and estrogen derivatives acting through their specific receptors [17, 19, 28]. On the other hand, no specific DHEA responsive nuclear receptor has been identified to date; and it is believed that the majority of physiological actions of DHEA should be attributed, depending on the tissue, to its conversion to either androgens or estrogens [19]. In THP-1 cells, we found that DHEA was converted to DHT, with its downstream metabolites 3α-diol and 3β-diol already appearing after 1 h of exposure. To support the role of androgens in DHEA effects, we found that by blocking 5α-reductase with finasteride, which prevents the conversion of testosterone to DHT as well as the conversion of 4-androstene-3,17-dione to 5α-androstanedione (which is then metabolized by 17β-hydroxysteroid oxidoreductase to DHT), DHEA-induced RACK1 expression can be prevented, implicating DHT as an effector androgen. The positive modulation of RACK1 expression induced by the androgenic effect of DHEA results in an increased response to LPS, as assessed by cytokine production. Compared to testosterone, DHT appears to be a more potent stimulus. At 1 nM DHT was already active, whereas testosterone at the same concentration was ineffective; this is in accordance with the higher stability of the DHT-AR complex [30].

Cadwallader et al. [31] have investigated hAR and hGR nuclear translocation in transiently transfected COS cells. Specific ligands induce rapid and robust nuclear translocation, without cross reactivity. For testosterone there was a slightly higher rate of transport calculated for the 50 nM dose than the 100 nM, which may explain the slightly lower activity of testosterone 100 nM that we observed compared to 10 nM (Fig. 2). This could be of physiological significance, since 10 nM is close to the physiological concentrations of androgens (0.7-3 nM in females and 10–50 nM in males). The loss of the immunostimulatory effect of DHEA by blocking or silencing the AR is consistent with the observation that AR ablation in myeloid cells tends to establish an immunosuppressive environment [32, 33].

## Conclusions

Overall, these data, together with the ability of physiologically relevant concentrations of testosterone and DHT to induce RACK1 expression, supports the notion that the metabolic transformation of DHEA to androgens and their binding to AR are required for DHEA-induced RACK1 expression and cell activation. Additional studies are required to fully understand the mechanism of AR action following DHEA treatment, especially the assessment of specific targets and/or coregulatory proteins recruited, which may also contribute to the effect of AR via interference and modulation of GR splicing isoform production, and in turn, their effects on the promoter region of GNB2L1 gene coding for RACK1 protein.

Hormones have an important role in homeostasis and function of the immune system. Sex hormones appear to have distinctive and exclusive roles in the development of the immune system and in shaping the immune responses [34]. It is intriguing to note that pharmacological concentrations of androgens have been reported to reduce immune cell activation (reviewed in ref [35]).

We identified DHEA conversion to androgens and binding to AR as necessary steps in DHEA-induced cell activation, and considering the opposite effect of cortisol, the data supports the existence of a complex hormonal balance between cortisol and androgens, which orchestrates RACK1 expression and monocyte activation, leading the way to novel therapeutic targets for immune modulation.

## Abbreviations

AR: androgen receptor
DHEA: dehydroepiandrosterone
DHT: dihydrotestosterone
DMSO: dimethyl sulfoxide
GR: glucocorticoid receptor
IL: interleukin
LPS: lipopolysaccharide
PBMCs: peripheral blood mononuclear cells
RACK1: receptor for activated kinase 1
SCR: scramble
TNF: tumor necrosis factor
